# Examining the components of freedom values in contemporary Chinese society

**DOI:** 10.3389/fpsyg.2024.1434161

**Published:** 2024-11-12

**Authors:** Yue Zhang, Heshui Huang, Jiao Tian

**Affiliations:** ^1^School of Journalism and Communication, Xiamen University, Xiamen, Fujian, China; ^2^School of Media and Communication, Guangxi Minzu University, Nanning, Guangxi, China

**Keywords:** freedom values, contemporary Chinese individuals, conceptual model, cultural context, societal well-being

## Abstract

**Introduction:**

This research paper delves into the structure and connotation of freedom values within contemporary Chinese society, taking into account the historical, societal, and cultural context.

**Methods:**

To achieve this, a multifaceted approach was adopted, integrating literature analysis, focus group interviews, in-depth interviews, and questionnaire surveys.

**Results:**

The study culminated in the development of a comprehensive conceptual model comprising five distinct dimensions: behavior within boundaries, independence without interference, autonomy with responsibility, rights with guarantee, and equality without domination.

**Discussion:**

This conceptual model successfully consolidates the prevailing consensus on freedom values among the Chinese populace, offering a robust framework for the propagation and nurturing of freedom within Chinese society. The insights gleaned from this research deepen our comprehension of freedom values and furnish valuable guidance for academics, policymakers, and practitioners keen on advancing freedom and societal well-being.

## Introduction

Freedom is an ancient and crucial subject within the realms of modern philosophy, politics, and communication. The pursuit of freedom as an ideology can be traced back to ancient Greek civilization. In the 17th century, Western society shaped the systematic value and political idea of freedom, laying the foundation for modern nations. This cornerstone value also resonates in China’s cultural heritage, evolving through its historical transformations. The 18th National Congress of the Communist Party of China (CPC) report integrated freedom into core socialist values as the primary societal value orientation. Since then, the study of freedom values has garnered significant attention and research focus within Chinese academia.

So, what exactly is freedom? Montesquieu highlighted in his book On the Spirit of the Laws that no other word possesses as many meanings and evokes such diverse impressions in people’s consciousness as freedom does (Cohler et al., 1989). In his book Two Concepts of Freedom, Berlin notes that throughout the history of thought, more than two hundred definitions of freedom have emerged (Berlin, 1969). These definitions are subject to varying interpretations by individuals, theories, and cultures. The complexity of the concept of freedom has given rise to numerous schools of thought and, to some extent, has posed challenges in the dissemination and cultivation of values.

According to research, there is a prevalent awareness and acknowledgment of freedom values among the public, indicating a societal consensus. However, a deficiency in understanding persists, which fails to effectively influence practical application (Li and Gao, 2021; Chen and Zhang, 2015). Even highly educated college students lack familiarity with the specific content of freedom values, finding them lacking in personal relevance or clarity, with uncertainties surrounding their practical implementation (Zuo and Feng, 2019; Tao, 2016; Li et al., 2015). In rural areas, the common scenario of not comprehending the essence of values often leads to misinterpretations, such as equating freedom with unrestricted autonomy and rejecting its true value (Liao and Qiao, 2016; Shen et al., 2017). Scholars have highlighted that the identification with the value of freedom among Chinese citizens may not necessarily originate from personal rationality and emotional inclinations but rather from straightforward or even misguided identification, making it challenging to address the existing divergences, uncertainties, or deviations in cognitive perception (Xing, 2018; Zhang and Qin, 2020).

In addressing the cognitive-practical dilemma prevalent in the current spread of freedom values, scholars have identified issues of emptiness, simplification, and doctrinalization in the dissemination process (Su, 2017; Chen and Liu, 2015). The politically inclined direction of the communication route contradicts the requirements of societal integration and daily life, impeding the effective spread of these values (Qiu and Zhou, 2017). The crux of the matter lies in the challenge of rectifying the dissemination of freedom values solely through broadening the dissemination scope and enhancing dissemination technology. The central issue is the disconnect between these values and public life, necessitating interpretation and absorption by individuals that resonate more closely with the masses. Only when individuals internalize the implications of the ideological framework and integrate them into their daily consciousness, communal mindset, and behavioral standards, can these values effectively influence and guide societal behavior (Plekhanov, 1962). Therefore, delving deeper and precisely conveying the core essence of freedom values forms the theoretical basis for enhancing their communicative impact.

Existing research on freedom’s value has predominantly focused on theoretical perspectives from disciplines such as philosophy, political science, and sociology, resulting in a substantial body of literature. However, these studies exhibit a pattern characterized by “three more and three less”: an abundance of theoretical inquiries alongside a scarcity of empirical investigations; a prevalence of expert assessment studies over public assessment studies; and a focus on exploring the importance of values rather than analyzing the challenges of dissemination and practical application. Although theoretical studies hold value, they are constrained in analyzing the present scenario and offering practical guidance, primarily due to overlooking the public’s subjectivity, presenting abstract content that is challenging for public comprehension, and lacking clear guidance on applying these values. A socially acknowledged framework for freedom is needed as a foundation for behavior within a social setting, shaping our comprehension of legal principles, rights, and societal norms (Campill, 2024).

Therefore, this study advocates that research on freedom values should transcend theoretical analysis, incorporate public voices, foster societal agreement, and offer contemporary Chinese interpretations of fundamental values. Within the Chinese context, this study utilizes literature analysis, detailed interviews, expert reviews, focus groups, questionnaire surveys, and statistical analysis to delve deeply into the essence and framework of Chinese people’s freedom values. The goal is to present interpretations of values that are more pertinent to people’s lives and easily comprehensible. By surmounting the limitations of prior studies, this research introduces novel perspectives on freedom values theory, proposes effective communication and nurturing strategies, and establishes a solid foundation for the practical application of freedom values.

### Theoretical background

The value of freedom encompasses the fundamental values, spirit, and concept of freedom, offering a direct reflection of individuals’ state of freedom and value orientation. Its definition is rooted in the understanding and application of freedom. The concept of freedom possesses a highly complex and ever-evolving connotation within the extensive stream of human thought. Through an extensive review of literature, we gain insights into people’s understanding and discourse on freedom and freedom values, encompassing perspectives from freedom within Chinese traditional culture, Western liberalism, Marxism, and socialism with Chinese characteristics.

The concept of freedom in traditional Chinese culture finds its origins in the interpretations of freedom put forth by Confucianism, Taoism, Zen Buddhism, and the School of Mind. Confucianism seeks moral freedom through the theory of “being kind to oneself” and emphasizes personal cultivation as a means to attain inner freedom, as encapsulated in Confucius’ saying that to do what I intend freely without breaking the rules. Taoism, Zen Buddhism, and the School of Mind all strive for spiritual freedom. In Taoism, freedom signifies acting in accordance with one’s natural disposition and achieving harmony with the Tao. Zen Buddhism draws from Confucianism’s teachings on mindfulness and incorporates the naturalistic philosophy of Laozhuang’s metaphysics. It views freedom as liberation from the limitations imposed by physical and mental desires. Lu Wang’s philosophy promotes the elimination of selfish desires and the restoration of one’s innate mind, thereby achieving the unity of knowledge and action through natural conduct (Wang, 2008). It becomes evident that freedom in the Chinese tradition is sought from within, through the cultivation of one’s heart and inner character. The pursuit of freedom is manifested in the self, the human heart (Qian, 2002). This introspective perspective on freedom underscores the importance of self-awareness and self-discipline.

Western liberalism has evolved through the developmental stages of classical liberalism, new liberalism (referred to as social liberalism in its later stage), and neoliberalism. Classical liberalism upholds the principle of “freedom without interference” and initially proposes concepts such as freedom of speech, belief, thought, publication, and economic freedom. Prominent figures associated with this ideology include Locke and Mill. Locke’s theory of freedom primarily focuses on the role of government, asserting that its responsibility is to safeguard individual freedom rather than restrict individual behavior (Locke, 1988). Mill’s theory of freedom expands to the societal level and introduces two principles regarding the relationship between individuals and society. The first principle is the principle of freedom from harm, affirming everyone’s right to be free from infringement. The second principle is the principle of fairness, highlighting that individuals in social life bear their own responsibilities and obligations (Mill, 1859). Neoliberalism, in comparison to classical liberalism, places greater emphasis on the significance of government intervention in achieving individual freedom and places increased focus on equality and social justice. Rawls explores freedom within the framework of human capabilities and develops a perspective that integrates freedom with equality. He asserts that freedom is an inherent structure of the system and suggests that the social structure should establish and adapt basic freedom based on the moral capacity of individuals (Rawls, 1999). Dworkin’s concept of “liberal equality” can also be seen as a response to this understanding of freedom. Neoclassical liberalism represents a revived version of 18th-century classical liberalism, emphasizing free competition rather than the pursuit of equality. It is evident that all forms of Western liberalism uphold the fundamental rights of individuals, with the primary distinction lying in the role of the government in societal affairs and the question of whether the pursuit of social justice should be embraced as a core value. In comparison to traditional Chinese liberalism, Western liberalism can be characterized as “outward-looking,” prioritizing the interactions between individuals and the state or society and striving to protect individual rights.

According to Marxist theory, freedom holds a central position and represents a fundamental value pursued by Marxism. The Marxist perspective on freedom asserts that freedom exists within the realm of tangible reality, rather than being an abstract concept confined to theory. It contends that genuine freedom manifests through the unrestricted and holistic growth of every individual. Marxism places the actuality of humanity as its foundation, emphasizing the integration of labor into the essence of human beings. It asserts that the examination of freedom must consider social relations and be actualized through productive practice, while simultaneously removing external societal constraints (such as alienated labor). Marx stated that “The nature of humanity is not an abstract quality confined to individuals; in reality, it encompasses the totality of social relations” (Marx and Engels, 1995a). He also argued that “Freedom does not reside in the illusion of detachment from natural laws but in comprehending these laws to systematically serve specific objectives” (Marx and Engels, 1995b). Marx posited that “The class character of human beings lies precisely in their capacity for conscious and voluntary action. It is due to their existence as class-conscious beings that their actions are truly free. Alienated labor distorts this relationship and subverts it” (Marx and Engels, 1979). Furthermore, through analyzing the inherent contradictions within capitalist society, Marx discerned the progression of human development into two distinct phases: the “Kingdom of Necessity” and the “Kingdom of Freedom.” He differentiated the degrees of freedom in human practical activities and emphasized the pivotal role played by the complete advancement of productive forces and the availability of free time. Marx highlighted their significance in enabling individuals to engage in autonomous pursuits and attain unrestricted personal growth (Marx and Engels, 2003). The Marxist notion of freedom diverges from the traditional Chinese concept that centers on the self and human emotions. Additionally, it serves as a critique and transcendence of Western liberalism, which is rooted in individualism and private ownership.

The concept of freedom promoted by socialist core values has its roots in traditional Chinese culture, while also drawing critical inspiration from Western liberalism and serving as an inheritance and evolution of the Marxist understanding of freedom. The freedom encapsulated in socialist core values pertains to the freedom of action, emphasizing the pursuit of tangible freedom rather than illusory notions. It calls for the translation of freedom into practical endeavors, becoming the autonomous and conscious activities of individuals. This freedom exhibits specificity, manifesting in various dimensions and levels, such as economic, political, moral, and ideological freedoms. Furthermore, it possesses historicity, as it is contingent upon social and historical circumstances within the socio-economic framework (Marx and Engels, 2001). Since China’s reform and opening-up, the concept of freedom has undergone a transformative journey, transitioning from a critique of “liberalization” to an emphasis on the holistic development of individuals, the establishment of a harmonious society, and the implementation of the rule of law. This evolution has led to the establishment of a socialist notion of freedom tailored to Chinese conditions for the new era (Kou, 2019). It is evident that the socialist concept of freedom represents a “synthesis of many provisions, “a “unity of diversity.” It is concrete, historical, and dynamic, closely intertwined with practical implementation in the actions of every citizen. Consequently, our research needs to account for the realities of China’s social development. We should gather public opinions, integrate consensus from diverse value judgments and interests, and seek the “greatest common denominator” of the concept of freedom. Simultaneously, we must concretize and contextualize the intricate and profound connotations of freedom derived from literature research. Additionally, we should explore people’s understanding of freedom within their daily lives, enabling the operationalization of the complex and abstract aspects of the concept. This will result in the creation of a communication model with clear dimensions and specific contents, which can effectively guide practice.

## Research method

Initially, a comprehensive collection of complex and diverse connotations of freedom values from various cultural backgrounds and historical periods is conducted through literature analysis. Subsequently, focus group interviews, in-depth interviews, and expert reviews are employed to delve into the specific content of freedom values among contemporary Chinese individuals, forming a comprehensive question bank to capture diverse perspectives on freedom. The study then utilizes the questionnaire survey method, accompanied by statistical techniques such as exploratory factor analysis and confirmatory factor analysis, to investigate and validate the composition of freedom values among the Chinese population.

### Research participants

#### Participants in focus group interviews

The participants in the focus group interviews were students and faculty members from a comprehensive university in China. A total of 42 participants representing different majors formed three focus groups, with an average of 14 participants per group. Among the participants, there were 16 males and 26 females, ranging in age from 18 to 63 years.

#### Participants in in-depth interviews

The participants in the in-depth interviews consisted of leading cadres, ordinary staff from government agencies and institutions, executives, general employees from enterprise units, as well as grassroots laborers and unemployed individuals. A total of 12 people were selected, including 6 men and 6 women, with an age range of 15–60 years.

#### Participants in program evaluation

The project was evaluated by 21 doctoral supervisors, as well as master’s and doctoral students from psychology, journalism and communication, political science, and philosophy. Among the evaluators, 6 were male and 15 were female, with an age range of 19–63 years.

#### Respondents to the liberal values questionnaire

The online questionnaire was distributed on the Credamo platform, resulting in 609 collected samples, with 499 samples deemed valid. The effective return rate of the questionnaire was 81.9%. Among the respondents, 169 (33.9%) were male and 330 (66.1%) were female. In terms of age distribution, 165 (33.1%) were aged 15–24 years, 241 (48.3%) were aged 25–34 years, 56 (11.2%) were aged 35–44 years, 25 (5%) were aged 45–54 years, 11 (2.2%) were aged 55–64 years, and 1 (0.2%) was aged 65 years and above. Regarding educational attainment, 6 (1.2%) had completed middle school or below, 26 (5.2%) had completed high school or junior college, 67 (13.4%) had completed college, 322 (64.5%) held a bachelor’s degree, and 78 (15.6%) held a master’s degree or higher. The 499 samples were randomly divided into two halves, with Sample 1 consisting of 279 participants used for exploratory factor analysis, and Sample 2 consisting of 220 participants used for validation factor analysis.

### Questionnaire development process

#### Generation of initial question items

The concept and structure of freedom values were explored based on the results of literature analysis, focus group interviews, and in-depth interviews. A total of 262 expressions related to freedom values were extracted through literature analysis. The focus group and in-depth interviews covered the following topics: (1) defining the concept of freedom, (2) understanding situations that people perceive as “unfree,” (3) exploring the perception of freedom in different social roles such as students, teachers, parents, children, leaders, subordinates, and consumers, and (4) investigating the understanding of freedom in various social contexts, including the family, workplace, and public arena. Three focus groups were conducted, and the opinions expressed in the third session overlapped with those of the previous two groups, resulting in no new expressions. Consequently, the focus group interviews were concluded, and in-depth interviews were conducted to gather additional information on the value of freedom from individuals outside the university. The focus group interviews and in-depth interviews, combined, yielded a total of 408 relevant expressions. In summary, these three sources provided a total of 670 descriptive statements.

The group reviewed and revised the original expressions based on the principles of semantic simplicity, semantic uniqueness, and connotation consistency. Expressions with unclear semantics were clarified, expressions with multiple layers of meaning were split, and expressions with similar semantics were merged. For example, the phrases “to express one’s opinion openly within the scope of the law without being attacked” and “to speak one’s mind boldly without being abused” were combined and revised to “being able to express any opinion as long as it does not violate the law.” Additionally, expressions that did not align with the connotation of freedom value, such as “one should be courageous and willing to give a hand to others,” were eliminated. These revisions resulted in 137 original items, which proceeded to the next round of item evaluation.

#### Formation of the initial questionnaire

The initial question pool underwent individual verification and evaluation by 21 project evaluators. Questions with excessive detail and specificity, such as “Users should not be forced to agree to the privacy agreement before using the software,” were excluded. Questions with independent meanings and broad representativeness were selected to be included in the questionnaire. Following this evaluation, the preliminary questionnaire consisted of 43 items (including 3 reverse questions) and was scored on a 5-point scale (1 = strongly disagree, 5 = strongly agree).

### Statistical methods

Statistical data processing and analysis were performed using SPSS 26.0 and AMOS 23.0 software.

### Analysis and findings

#### Item analysis

Sample 1 was divided into two subgroups based on the total score: the high subgroup and the low subgroup. The first 27% of scores were categorized as the high subgroup, while the subsequent 27% were classified as the low subgroup. An independent samples t-test was conducted to compare the scores of the high and low subgroups. The results indicated a significant difference in scores between the two groups for all initial items (*p* < 0.05). Furthermore, the correlation coefficients between each item and the total score were calculated. Six items (Q1, Q31, Q35, Q37, Q41, Q42) with correlation coefficients below 0.30 were excluded. The remaining 37 items exhibited correlations with the total score ranging from 0.307 to 0.574 (*p* < 0.001).

### Construct validity

#### Exploratory factor analysis

Exploratory factor analysis was performed on the 37 items using Sample 1. The Kaiser-Meyer-Olkin measure of sampling adequacy (KMO = 0.862) and Bartlett’s test of sphericity (*χ*^2^ = 2767.683, *p* < 0.001) indicated that the data were suitable for factor analysis. Principal component analysis was employed to extract the factors, adhering to the principles of appropriate item attribution. Five factors with eigenvalues greater than 1 were extracted, resulting in a total of 16 items. The factor loadings of each item ranged from 0.59 to 0.79, explaining 58.16% of the total variance. Factor 1, named “Behavior without Interference,” comprised 4 items that reflected an individual’s freedom of behavior within the bounds of the law. Factor 2, named “Independence without Interference,” consisted of 4 items that represented an individual’s independence in refraining from interfering with others and being free from interference. Factor 3, named “Autonomy and Responsibility,” included 3 items and related to individuals’ sense of responsibility for their own choices. Factor 4, named “Rights are Guaranteed,” comprised 3 items and focused on the assertion that individuals’ rights in society are safeguarded. Factor 5, named “Equality is not Dominated,” consisted of 2 items and pertained to the willingness to foster equal interaction among different generations and hierarchical positions. Please refer to Table 1 for detailed information.

**Table 1:** Results of exploratory factor analysis on the components of Chinese people’s freedom values (*N* = 279).

	Component				
Factors and items	f 1	f 2	f 3	f4	f 5
Factor 1: behavior within boundaries					
1. Legally permissible, individuals can acquire and possess any item.	0.777				
2. As long as it is not illegal, what to eat is a personal choice and others cannot interfere	0.765				
3. Non-illegal sexual behavior should not be restricted.	0.726				
4. Any speech can be expressed, as long as it does not violate laws or regulations.	0.686				
Factor 2: independence without interference					
1. Without permission, one must not intrude into others’ privacy.		0.724			
2.Imposing one’s own will on others should be avoided		0.689			
3. One should have the courage to refuse illegal requests from others.		0.607			
4. Interfering with others’ interests and hobbies should be avoided.		0.585			
Factor 3: autonomy with responsibility					
1. Personal destiny should be in one’s own hands.			0.771		
2. One should take responsibility for any choices they make.			0.704		
3. It is important to choose one’s own path according to personal desires.			0.671		
Factor 4: rights with guarantee					
1. People should be allowed to participate in various legal activities.				0.761	
2. People should be allowed to join legal groups to their own will.				0.707	
3. Legitimate religious beliefs should be respected				0.648	
Factor 5: equality without domination					
1. Elders should not demand that younger generations must do something.					0.791
2. Leaders should not exploit their position of authority to make employees do personal tasks.					0.734

#### Validation factor analysis

**Figure 1:**
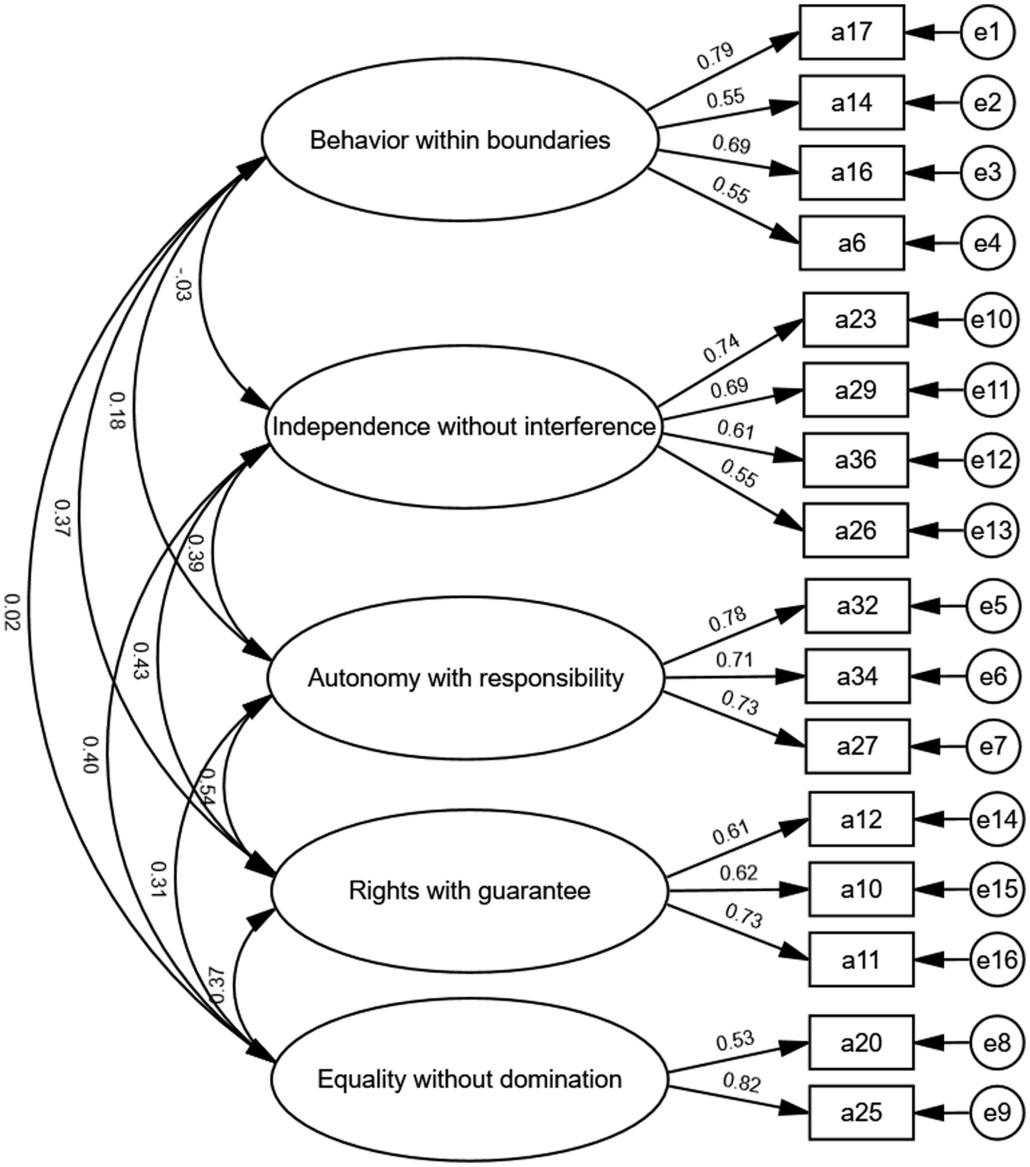
The five-factor structural model of Chinese people’s freedom values.

To assess the reliability of the aforementioned five-factor structure, a validation factor analysis was performed using Sample 2. The results indicated a good fit for the five-factor model, as evidenced by the following specific indices: *χ*^2^/df = 1.456, CFI = 0.950, TLI = 0.936, IFI = 0.951, and RMSEA = 0.046. The loadings of each item on their respective factor ranged from 0.534 to −0.789 (see Figure 1).

**Figure 2:**
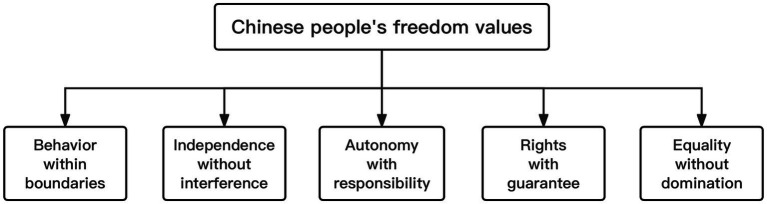
Structure of Chinese people’s freedom values.

Reliability and validity tests were further conducted. The Cronbach’s alpha coefficient of the revised total scale was 0.75, and the Cronbach’s alpha coefficients of the five factors were 0.74, 0.73, 0.78, 0.69, and 0.60, respectively. All the factors had reliabilities greater than 0.6, which is considered acceptable. The average variance of variance (AVE) for each latent variable exceeded 0.4, and the combined reliability (CR) was above 0.6, indicating satisfactory convergent validity for the five-factor structure. After the discriminant validity test, we observed a significant correlation (*p* < 0.01) between the five factors: “Behavior within boundaries,” “Independence without interference,” “Autonomy with responsibility,” “Rights with guarantee,” and “Equality without domination.” Furthermore, the absolute values of the correlation coefficients were less than 0.5 and all lower than the square root of the corresponding AVEs, demonstrating ideal discriminant validity of the scale (Figure 2).

## Conclusion and discussion

This study utilized nationwide survey data and employed a combination of literature analysis, interviews, and questionnaires, as well as exploratory factor analysis and validation factor analysis techniques. The objective was to develop a model of Chinese values of freedom. Through these methods, a five-factor structure emerged, consisting of behavior within boundaries, independence without interference, autonomy with responsibility, rights with guarantee, and equality without domination. Subsequently, a comprehensive five-factor model representing Chinese freedom values was established, encompassing the dimensions of “behavior within boundaries,” “independence without interference,” “autonomy with responsibility,” “rights with guarantee,” and “equality without domination.”

“Behavior within boundaries” refers to the notion that individuals enjoy complete freedom in their daily conduct while adhering to legal limitations. This dimension encompasses various aspects, such as the freedom to purchase and possess goods, engage in eating and drinking, sexual freedom, and freedom of speech. These elements highlight the universality of physiological and psychological needs inherent in both men and women, as dictated by human nature. Human nature, which serves as the foundation for all value judgments and codes of conduct, underscores the importance of comprehending it when discussing values and behavioral norms. Contemporary psychological science theories reveal that needs rooted in human nature form the objective psychological basis for the development of moral principles (Schwartz and Bilsky, 1987). Confucianism espouses the theory of moral freedom through self-benevolence, wherein moral behavior is consciously aligned with human nature. This approach aims to enhance moral cultivation, enabling individuals to naturally exhibit behavior that aligns with their own will while adhering to social propriety. Nevertheless, it must be acknowledged that this ideal state remains elusive, as even Confucius himself acknowledged, stating, “At seventy, I could follow my heart’s desire without overstepping the boundaries.” Confucius believed that genuine freedom exists within the boundaries of ritual and music. According to Rawls, a contemporary political philosopher, freedom is a systemic structure governed by norms that outline rights and obligations, and it should be discussed within the framework of legal limits (Rawls, 1999). Due to individual differences, each person possesses distinct needs and interests. Without appropriate regulation, individuals, driven solely by their self-interest, would be unable to restrain their pursuits. Thus, social norms and legal constraints are necessary to regulate the diverse needs and interests of individuals, striking a balance between collective interests. The freedom of human behavior should be guided by reason throughout one’s actions, way of life, and self-preservation (Kisner, 2011). The findings regarding the dimension of “behavior within boundaries” emphasize the fundamental importance of respecting human nature while cultivating the concept of civil liberties. Simultaneously, it is crucial to define human liberties within legal boundaries to ensure that freedoms are realized within those limits.

“Independence without interference” signifies that individuals have the capacity to think and act autonomously, free from external intervention, while also refraining from impeding the freedoms of others. This encompasses various aspects, such as protection against unlawful intrusions into one’s privacy, freedom from the imposition of one’s will, the ability to pursue personal hobbies and interests without interference, and the right to reject unlawful requests from others. The concept of freedom from interference by others aligns with Berlin’s notion of negative freedom, where freedom is defined as the absence of interference by external agents. The breadth of freedom expands as the scope of non-interference widens (Berlin, 1969). In this dimension, we observe that Chinese people primarily seek negative freedom, focused predominantly on freedom from societal and interpersonal interference. This differs significantly from Western liberalism, which perceives the law as the primary impediment to freedom. Chinese society, in contrast, can be characterized as a vernacular society, functioning as a “society of acquaintances,” where individuals’ emotions are deeply rooted in their social lives (Fei, 2010; Blau, 1964). Feelings of joy, pain, freedom, or constraint are derived from interactions with others. In Chinese society, freedom holds substantial value as a regulatory standard governing interpersonal relationships and the relationship between individuals and society. It safeguards individual thought, preserves individuality, and upholds individual dignity. The dimension of “independence without interference” and its associated aspects suggest that fostering the concept of civil liberties necessitates a focus on transmitting values of mutual respect and non-interference. Each individual should strive to be true to themselves, emphasizing self-discipline over external control.

“Autonomy with responsibility” encapsulates the notion of autonomous motives, independent choices, self-reliance, and self-accountability. It grants individuals the freedom to chart their own path, pursue personal development, strive for self-defined ideals, and assume responsibility for their choices. This dimension primarily emphasizes positive freedom, characterized by the pursuit of “initiative” and the individual’s desire to be the master of their own destiny. Decisions and choices are driven by one’s own will, rather than external influences (Berlin, 1969). Freedom transcends the mere lack of constraints, constituting a intricate interplay among agency, responsibility, and social ties (Ernø and Birk, 2024). When individuals possess autonomous thinking and self-determined behavior, they experience a state of active freedom. The ability to exercise choice and be accountable for one’s decisions reflects the widely accepted notion that freedom is the union of rights and responsibilities. During in-depth interviews, we identified an implicit underpinning behind the content related to this dimension—a “concept of competency-based freedom.” Freedom becomes intertwined with the idea of competence. Several interviewees, when discussing the freedom to make independent choices and exercise self-determination, further acknowledged that “the freedom to choose does not necessarily guarantee comprehensive and equitable development; it is constrained by an individual’s abilities and living conditions.” However, having the freedom to choose provides each individual with greater opportunities to pursue their goals. It also serves as a motivational force, encouraging individuals to exert effort and enhance their willingness and ability to take responsibility. As for the outcomes, individuals expressed a commitment to “do their best and trust in a higher power.” Amartya Sen has elaborated on this perspective, asserting that freedom, particularly substantive freedom, encompasses viable capabilities. This entails considering not only the basic goods possessed by individuals but also the relevant personal characteristics that facilitate the transformation of these goods into tangible achievements (Sen, 1999). Values of equality and justice are integral to this consideration. The findings regarding “autonomy with responsibility” underscore the need to emphasize the development of autonomy and responsibility when fostering the concept of civil liberties. It is crucial to focus on the process of goal selection and the pursuit of ideals, actively exert subjective efforts, and strive to enhance personal abilities.

“Rights with guarantee” centers around citizens’ demands for assured political rights and freedoms. This includes the freedom to engage in lawful activities, join legal organizations, and practice their religious beliefs. China has established a comprehensive legal system to safeguard human rights, with political freedoms of citizens recognized and protected by the Constitution. Consequently, people’s rights to political freedoms are fully respected, facilitating their smooth realization and ensuring freedom from unlawful interference and intrusion. The concept of freedom within the framework of the law is vital. As the founder of Western liberalism, Locke, once stated, “The purpose of the law is not to abolish or restrict freedom, but to safeguard and enhance it … For freedom implies freedom from the bondage and exploitation of others, and where there is no law, there can be no such freedom” (Locke, 1988). Marx similarly asserted that “Freedom recognized by law exists as a state of law. The law is not a means of suppressing freedom, just as the law of gravity does not prevent motion” (Marx and Engels, 1995a). Abstract freedom takes on tangible form when elevated to a legal right by the law. The realization of freedom becomes concrete, and its enjoyment can be truly experienced when individuals exercise the rights to freedom granted and protected by the law. “Rights with guarantee” underscores the significance of legal protection in ensuring citizens’ realization of freedom. In the process of promoting the concept of civil liberties, it is imperative to simultaneously advance the development and integrity of the legal system. Additionally, it is essential to enhance public awareness of the rule of law through education and publicity efforts, thus strengthening citizens’ understanding of legal norms and safeguarding the fulfillment of their rights to freedom.

“Equality without domination” encapsulates the notion of equal and non-dominating freedom, which pertains to liberation from the control of others, such as paternalistic elders or authoritative leaders. This dynamic involves a hierarchical relationship characterized by unequal social status and a dominant power held by the “other” over the individual. The “other” can take the form of a patriarchal elder or a tyrannical leader, and their dominance creates an imbalance in social status and exerts control over the individual. This dominant power arises from disparities in social status, accompanied by the capacity for arbitrary interference, which poses a direct threat to the freedom of the dominated individual and inflicts harm on their interests (Pettit, 1996). However, it is an objective reality that people possess varying social statuses, influenced by factors such as seniority, position, wealth creation, and contribution. These differences result in variations in voice, resource mobilization, and influence. A rational social structure forms the foundation for the functioning of economic and social life, and it is neither feasible nor desirable to eliminate disparities in social status. Therefore, the pursuit of equal and non-dominating freedom by citizens fundamentally rests on rejecting arbitrary interference and the harm it inflicts on the dominated. Achieving this goal necessitates the cultivation of moral values and the establishment of a robust rule of law. Strengthening civic morality enables individuals from different generations, identities, and social statuses to engage with one another respectfully. Enhancing safeguards for the rule of law addresses the problem of uncontrolled exercise of power while also demanding that citizens assume responsibility for upholding the rule of law. This involves placing trust in the rule of law, actively holding officials accountable through their actions, and displaying zero tolerance for the abuse of power and corruption. The dimension of “equality without domination” suggests that, in the process of fostering the concept of freedom, it is essential to advocate for equal interaction and mutual respect. Additionally, there is a need to reinforce citizens’ moral consciousness and their adherence to the principles of the rule of law. By doing so, arbitrary domination and the abuse of power can be rejected, contributing to the realization of a society based on freedom.

## Theoretical and practical implications

The primary contribution of this study lies in constructing a systematic and lucid framework of Chinese people’s freedom values through empirical research methods. In comparison to previous studies, this research embodies significant methodological innovation and broadens practical applications based on theoretical inquiry.

Historically, previous studies have predominantly focused on theoretical inquiry, which has been overly abstract and conceptualized, lacking in operationalization and empirical testing of the essence of freedom values. Consequently, the dissemination of freedom values often remains at the level of reduction to slogans, making it challenging for the general public to comprehend and accept, and even more arduous to play a guiding role in societal practices. To overcome this limitation, this study adopts innovative strategies: Firstly, practical orientation: the study is grounded in the specific context of Chinese society, concentrating on the nuanced understanding and requirements of various social groups regarding freedom values, ensuring that the research outcomes possess both practical significance and value. Secondly, integration of public opinion: Through extensive questionnaire surveys, focus group interviews, in-depth interviews, and other empirical methods, the study collected and analyzed the public’s perceptions, attitudes, and behaviors toward the value of freedom to ensure broad-based representation in the research. Thirdly, empirical research: employing a blend of quantitative and qualitative methodologies, the study delved into the essence of freedom values, offering robust data support. Fourthly, tailored communication and cultivation strategies: based on the developed framework for Chinese people’s freedom values, the study provides specific strategies for communication and nurturing, offering a coherent guidance for policy formulation and societal application.

By introducing these methodological innovations and conducting thorough empirical research, this study effectively addresses the limitations of existing studies, introduces new perspectives and tools for understanding and disseminating freedom values, and holds significant academic and practical value.

## Conclusion, limitations and future research

In summary, this study reveals the contemporary Chinese concept of freedom as a synthesis and progression of traditional Chinese, Western liberal, and Marxist ideas of freedom. The Chinese understanding of freedom is built upon constraints, self-discipline, legal guarantees, autonomous development, responsibility, and an inherent connection to the principles of equality and justice. By elucidating the connotation and structural characteristics of this contemporary Chinese concept of freedom, we provide a framework for deepening the dissemination of socialist core values pertaining to freedom, as well as a reference for fostering freedom within Chinese society.

Nevertheless, it is important to acknowledge the limitations of this study, as it represents the first empirical exploration of the structural model of Chinese people’s freedom values. Improvements can be made in future research endeavors to address these shortcomings. Due to constraints in terms of time and funding, the size and composition of the research sample were restricted, with a notable overrepresentation of female, young, and highly educated participants. These factors may have influenced the conclusions drawn. Consequently, future studies should aim to enhance the reliability and stability of the structure of Chinese people’s freedom values by optimizing sample size and composition through more extensive sampling.

The universal pursuit of freedom as a spreading value contributes to individual fulfillment and societal inclusivity. Building upon this new framework, future research can expand in the following directions: enhancing the international communication of freedom values and establishing a discourse system conducive to global exchanges; engaging in cross-cultural communication and comparative studies to foster consensus on values across diverse cultural contexts; developing measurement scales and employing quantitative research methods to evaluate the impact and evolution of values. In conclusion, the exploration of freedom values holds significant practical importance and warrants in-depth focus and study.

## Data Availability

The original contributions presented in the study are included in the article/supplementary material, further inquiries can be directed to the corresponding author.
